# Superficial plantar foreign body mimicking plantar fasciitis: A case report

**DOI:** 10.1016/j.ijscr.2025.112128

**Published:** 2025-10-27

**Authors:** Huabin Chen, Feng Zhang

**Affiliations:** a Health Management Center, Taizhou Hospital of Zhejiang Province, Affiliated to Wenzhou Medical University, Linhai, Zhejiang, China; b Taizhou Hospital of Zhejiang Province Affiliated to Wenzhou Medical University, Linhai, Zhejiang, China

**Keywords:** Foreign bodies, plantar fasciitis, Foot injuries, Diagnosis, Differential

## Abstract

**Introduction:**

Penetrating foot injuries often involve retained foreign bodies (FBs), Although deep retained FBs are well recognized, superficial metallic FBs embedded specifically within the stratum corneum and mimicking plantar fasciitis are rarely reported and can be easily misdiagnosed.

**Case presentation:**

A 55-year-old male physician experienced 2 weeks of left plantar pain during activity, self-diagnosed as plantar fasciitis，never had a medical check-up at the hospital. Due to the patient's own professional sensitivity as a doctor，a 1 mm black spot was noted on examination by himself，he decided to use a needle to prick this spot. A 2 mm stainless steel fragment was removed from the this black spot of stratum corneum, with immediate pain resolution.

**Clinical discussion:**

Superficial FBs embedded in thick plantar stratum corneum may avoid detection while causing weight-bearing significant weight-bearing pain due to dermal compression. This case highlights FB retention as a critical differential diagnosis for plantar pain, particularly post-trauma. The initial misdiagnosis occurred due to symptom similarity and the patient's prior experience; it underscores the importance of meticulous history and physical examination focusing on potential entry points or subtle skin changes, even if there is no significant history of foreign body punctures. It emphasizes the importance of a meticulous medical history and meticulous physical examination, and timely visits to a clinical specialist for chronic plantar pain.

**Conclusion:**

Plantar FB retention, including within the superficial stratum corneum, should be considered early in the differential diagnosis of unexplained foot pain, especially after potential penetrating injury. Thorough physical examination and history are paramount for diagnosis. This case highlights the importance of considering superficial retained foreign bodies in patients presenting with chronic plantar pain.

## Highlights

• Novel presentation of metallic FB retained within plantar stratum corneum mimicking fasciitis
• 2-mm FB hidden in 1.5-mm thick stratum corneum causes weight-bearing pain
• Office-based needle probing for rapid diagnosis/removal of plantar FBs, avoids imaging
• Reveals unique pain mechanism of superficial FBs compressing into dermis during load
• Emphasizes FB screening in initial differential diagnosis of plantar pain

## 1 Introduction

### 1.1 Background

The foot is an important locomotor organ of the human body. Plantar heel pain represents one of the most common foot complaints in adulthood, with potential etiologies including bone disorders, soft tissue pathology, nerve entrapment, or systemic diseases [1,2]. With the exception of trauma, the most frequent cause of chronic pain in the lower surface of the heel is plantar fasciitis [[3], [4], [5], [6], [7]].

Puncture wounds to the plantar surface of the foot are common injuries seen in emergency departments. These wounds generally occur in persons of all ages and in healthy patient populations. Plantar puncture wounds of the foot can be complicated by cellulitis, lymphangitis, soft tissue abscess, osteochondritis, and osteomyelitis [8]. It can be stabbed by various foreign bodies. Metallic foreign bodies include iron nails, iron wires, sewing needles, and broken injection needles. Non-metallic foreign bodies include glass, fragments of porcelain pieces, broken stones, gravel, thorns of bamboo and wood, and broken fishbone spines [9]. According to Bairş Polat: Metal objects can remain in tissues without causing any complications, but wooden particles can cause infections and consequently should be extracted [10].

This case report has been reported in line with the SCARE 2025 criteria [11].

## 2 Case presentation

### 2.1 Chief complaint

A 55-year-old male physician with extensive experience in outdoor sports and familiarity with common foot conditions presented with a two-week history of sharp, activity-dependent pain localized to the lateral forefoot of his left foot (pinprick-like pain localized to the lateral forefoot, which occasionally radiated diffusely across the plantar surface during weight-bearing, and the pain disappears when the foot is lifted or rested, and the symptoms closely mimicked those of plantar fasciitis, which is difficult to distinguish).

The symptoms began immediately after an episode of forcefully stepping onto an uneven rock surface during mountain climbing. The patient recalled a sudden sharp pain from the impact of a pointed rock against his left plantar foot, which subsided shortly after. He initially attributed the pain to a combination of blunt force injury and possible soft tissue strain, rather than a definitive fascial tear, which would typically warrant imaging caused by the impact and the jumping motion. Subsequently, he developed characteristic stabbing pain exclusively during weight-bearing activities, which also radiated towards the midfoot region. Although the location of this pain (forefoot) differed from his previous episodes of plantar fasciitis, which were typically situated under the heel or typically located in the heel or arch area, and despite thorough self-inspection revealing no visible shoe damage or retained ground debris, the persistent symptoms led him to self-diagnose this episode as recurrent plantar fasciitis (attributing it to tissue irritation and localized inflammation secondary to the rock impact and strain). For two weeks after the injury, the patient rarely went out, mainly stayed at home to rest, and was very careful when walking. Not exercising for two weeks can lead to weight gain and mental health problems. The patient based on prior experience with self-limiting foot complaints, initially opted for self-management rather than immediate clinical consultation. There is no specific treatment for fasciitis.

### 2.2 Past medical history

Prior episodes of plantar pain diagnosed as plantar fasciitis resolved spontaneously with rest. Drug History and Allergies: no.

### 2.3 Physical signs

General condition normal. A 1 mm non-protruding black spot on the left forefoot lateral aspect showed no tenderness or mobility limitation. Pain occurred only during weight-bearing activities.

### 2.4 Auxiliary examination

No imaging performed (foreign body analysis done post-removal).

### 2.5 Diagnosis

Foreign body retention in left plantar skin.

### 2.6 Treatment method

A 1 mm black spot was noted on examination by himself，patient decided to use a needle to prick this spot. Under aseptic precautions, a medical blood collection needle was used to extract a 2 mm arched stainless steel fragment embedded horizontally in the stratum corneum. The operator was the patient himself, who was a highly experienced surgeon.

## 3 Result

**Fig. 1:**
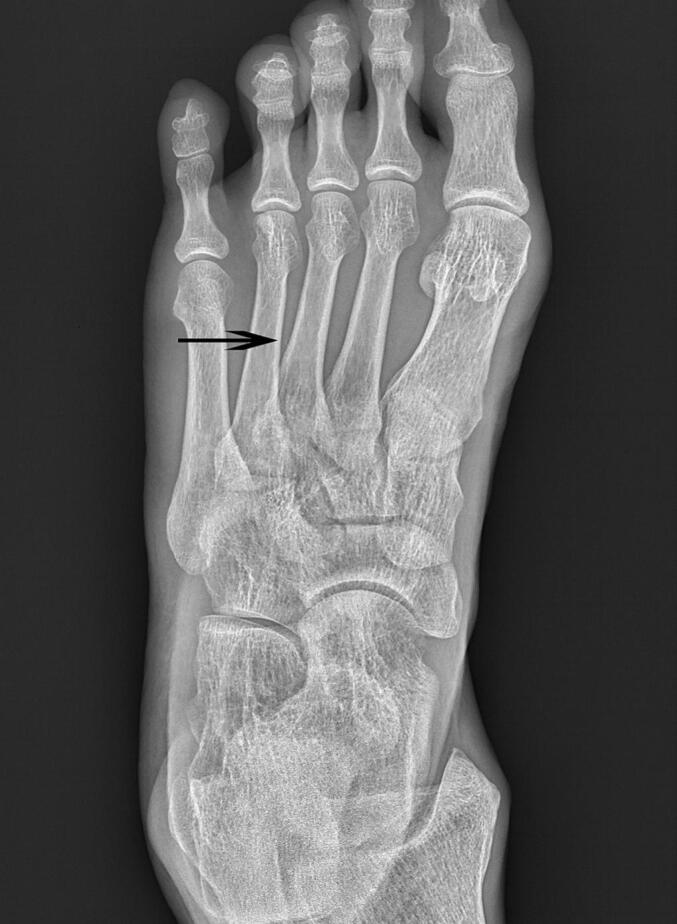
An arched stainless steel wire about 2 mm long is located in the middle section between the third and fourth metatarsal bones.

**Fig. 2:**
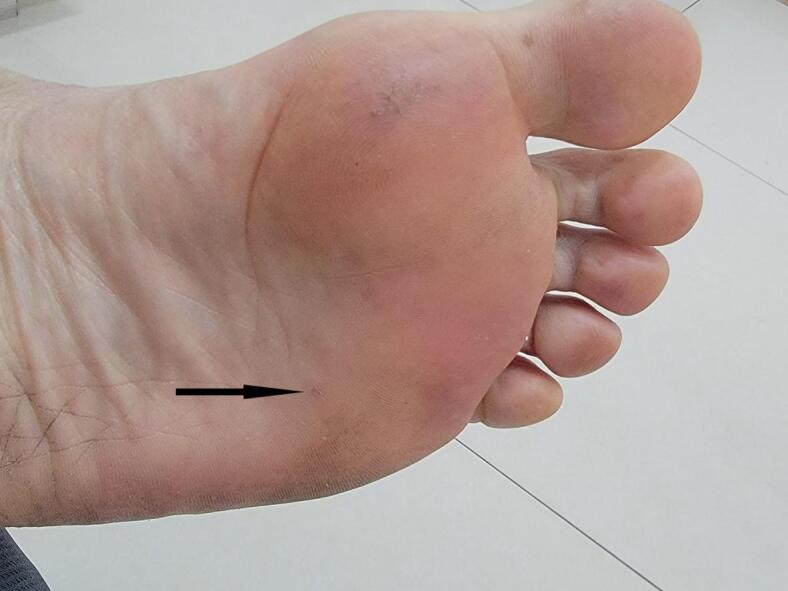
The photograph of the sole of the foot after the operation. The location pointed by the black tip is where the foreign body was located.

Complete pain resolution occurred immediately post-extraction. The minor epidermal defect healed rapidly (Fig. 1). He acted freely immediately after the operation, and there were no sequelae after the review a few days after the operation (Fig. 2).

## 4 Discussion

Diagnostic imaging examinations are rarely used for patients with plantar fasciitis. Clinically, there are many diseases that may produce clinical features highly similar to those of plantar fasciitis, so misdiagnosis is likely to occur. Plantar fasciitis remains a well-studied topic, most likely owing to the high prevalence of the condition and the lack of clarity regarding how best to treat the condition [12]. The patient based on prior experience with self-limiting foot complaints, initially opted for self-management rather than immediate clinical consultation, plantar fasciitis does not need auxiliary examination, and there is no special treatment, mainly rest and physiotherapy.

Plantar FB retention and fasciitis are common orthopedic pain sources. The plantar fascia supports the foot arch and absorbs ground reaction forces. Prolonged stretching or impact may cause aseptic inflammation (“plantar fasciitis”) – a prevalent sports injury [13]. However, numerous conditions mimic its symptoms, including:

• Soft tissue chondroma [14]
• Metastatic carcinoma [15]
• Atypical gouty arthritis [16]

Plantar heel pain in adulthood is the most common problem of the foot, and can be provoked by bone, soft tissue, nerve, or systemic disease. Although accurate diagnosis and appropriate management are important, distinguishing the various causes of highly similar symptoms is difficult. Plantar heel pains from skeletal prob- lems are caused by calcaneal stress fracture, apophysitis of the calcaneus (Sever's disease), osteomyelitis, or in- flammatory arthropathy. Soft tissue pathology includes fat pad atrophy (FPA) or contusion, plantar fascia rup- ture and plantar fasciitis (PF). Heel pain may be induced by neural causes such as entrapment or compression of the first branch of the lateral plantar nerve (Baxter's nerve), medial calcaneal branch of posterior tibial nerve, or nerve to abductor digiti quinti muscle. Other neural causes include S1 radiculopathy, tarsal tunnel syndrome, and peripheral neuropathy [17]. This case uniquely adds superficial stratum corneum FB retention to this differential diagnosis list.

**Fig. 3:**
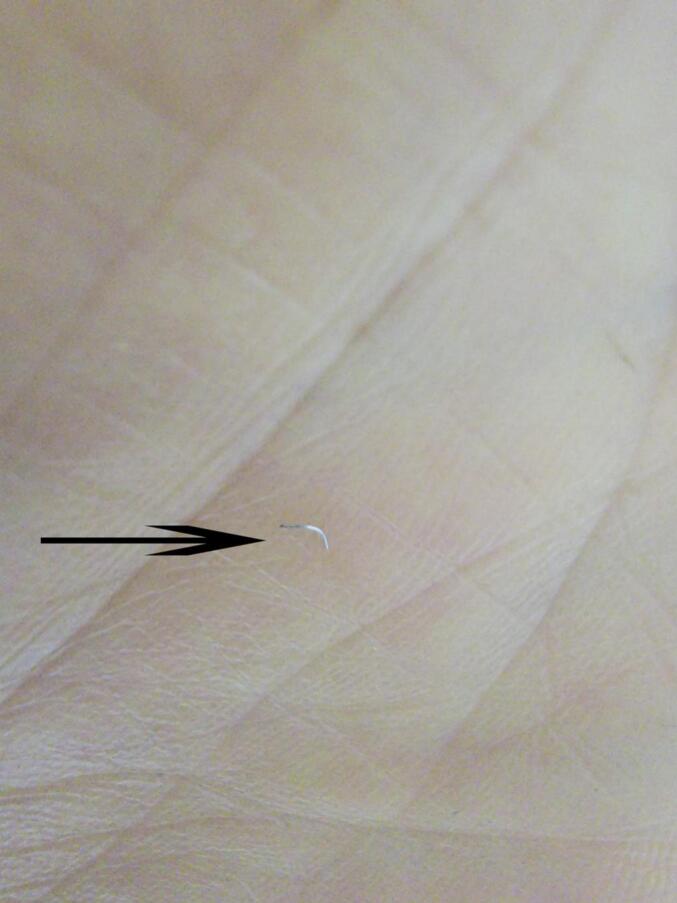
This arched stainless steel wire, which is the foreign body in the sole of the foot, is about 2 mm long and the height of the arch is approximately 1 mm.

**Fig. 4:**
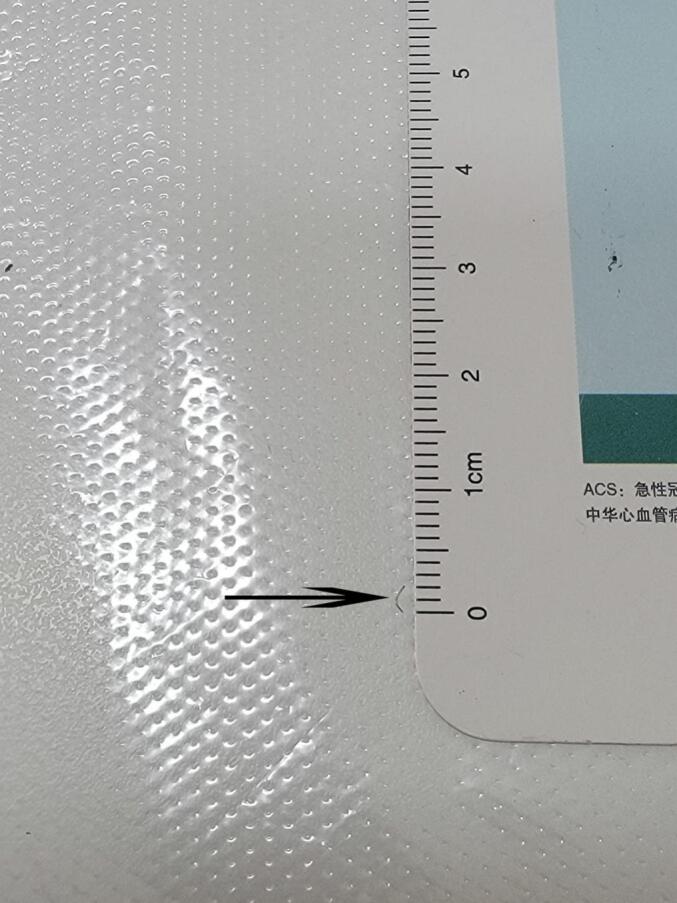
During exercise, the sole of the foot rubs against the ground, and the tiny metallic foreign body sandwiched between the skin and the ground is pierced into the stratum corneum of the plantar skin.

Given the acute post-traumatic onset and focal nature of the symptoms, the initial consideration was for residual bruising or necrotic stratum corneum from the impact two weeks prior. The patient questioned whether subcorneal hematoma might be causing the pain. Crucially, foreign body retention was not suspected initially. Therefore, no diagnostic imaging was obtained prior to intervention. Under aseptic precautions, office-based needle exploration was performed using a medical blood collection needle to investigate the area beneath the suspicious spot. This exploration revealed and successfully extracted a 2 mm arched, sharp, irregularly spiral-shaped, and elastic stainless steel fragment embedded horizontally within the stratum corneum layer (Fig. 3). The procedure was performed by the patient himself, leveraging his surgical expertise (See Fig. 4.).

The foot's unique anatomy (thick stratum corneum, muscle/tendon density) facilitates FB migration. Optimal FB management involves removal within 6–8 h post-injury [18], though presentation timing varies significantly, Once a foreign body is retained in the foot, the presentation varies according to the size, nature, location, and contamination [19].

It is difficult to determine soft tissue foreign bodies clinically, and the most common cause of medical disputes is that the sole of the foot is the most common part of foreign body residue, and it is more difficult to detect and remove foreign bodies due to dense tissue [20]. The foot's unique anatomy, characterized by a thick stratum corneum (up to 1.5 mm on the plantar surface [21]) and dense underlying tissue, can facilitate FB migration or deep embedding. While deep retained FBs are well-documented complications of penetrating injuries, superficial FBs confined strictly within the stratum corneum are rarely reported and easily overlooked. The author also learned that for the patient, prolonged outdoor swimming caused the skin at the bottom of the feet to remain submerged in water for a long time, which might lead to the water absorption and expansion of the stratum corneum on the sole of the foot, making it softer. Before the injury, the patient had just finished swimming in the mountain reservoir.

This case demonstrates four critical points:

1. Concealment: Small metallic FBs (≤1 mm in height) can become embedded within the thick plantar stratum corneum without penetrating the vascular or neural dermis, evading immediate detection and avoiding overt inflammatory signs. While other foreign objects penetrate directly, they gain more energy, and as a result, the foreign objects will reach deeper tissues. Both of them are prone to being missed or misdiagnosed.
2. Mechanism of Pain: Weight-bearing forces compress the embedded FB downwards, effectively driving it against the underlying, more sensitive dermal structures, triggering localized pain. At the time of the injury, it might have pierced the dermal layer, causing pain, and then returned to the epidermis, resulting in a small amount of blood.
3. Diagnostic Challenge: The absence of a clear entry wound or history of specific injury, combined with pain in the fascial area throughout the plantar of the foot, readily leads to misdiagnosis as plantar fasciitis, especially in the context of recurrent symptoms.
4. Diagnostic Approach and the Needle Probing Technique
  The feet and hands were the most affected parts of the body, since they are open to external penetrant injuries. The pairing of acral skin with a scalpel blade is a common procedure. This technique can be used to remove a foreign body from any physician's office. The use of this method reduces pain and complications with foreign body removal and encourages patients to seek professional assistance sooner. This is an efficient, effective, and acceptable method that is simple and less intimidating to patients. There is no numbing or incising involved, reducing stress on the physician and the patient. Patient satisfaction is high with this procedure. Foreign bodies of acral skin are very common, and this method proves to be simple and effective [22]. The diagnostic needle probing used by the author of this article is very effective. The principle is the same, and it is suitable for use in the office, at home, especially outdoors.

When a superficial FB is suspected based on localized point tenderness, careful inspection under magnification and palpation for subtle irregularities are essential. In this specific case, the FB was fortuitously identified and removed by the patient through self-examination and probing with a sterile needle. While office-based needle probing therapeutically facilitated removal after the FB was visualized, its role as a primary diagnostic tool in the absence of FB suspicion is not supported by standard practice and carries risks (e.g., infection, driving the FB deeper, nerve/vessel damage). Imaging modalities, particularly ultrasound (highly sensitive for superficial FBs) or radiography (for radiopaque objects like metal), are the recommended first-line investigations for suspected FBs when physical examination is inconclusive or the history is unclear. MRI, However, it is expensive and the waiting time is long while excellent for soft tissue detail (Metal cannot be imaged.), is often reserved for complex cases or deeper suspected pathology.

## 5 Conclusion

Unexplained foot pain warrants FB suspicion. Thorough history/examination prevents delayed diagnosis, particularly after penetrating injuries. This case highlights the importance of considering superficial retained foreign bodies in the differential diagnosis of chronic plantar pain. Superficial foreign objects, such as Metal shavings, stainless steel fragments, Sand and gravel, wood chips，etc., may unknowingly find their way into shoe soles or socks, causing invisible damage while walking, running, making it difficult to prevent.

If you have sudden plantar pain while walking outdoors, first carefully examine the soles of your feet to identify whether there is a foreign body on the plantar and determine whether the foreign body needle picking is the fastest and easiest solution.

### 5.1 Strengths and limitations

Strengths:

Novelty: This report highlights the rare occurrence and diagnostic challenge of a superficial metallic FB retained solely within the plantar stratum corneum, masquerading as plantar fasciitis.
Clinical Relevance: It emphasizes a commonly overlooked differential diagnosis for a very common condition (plantar heel pain).
Educational Value: It illustrates the consequences of delayed FB diagnosis and reinforces thorough history/exam.
Therapeutic Technique: Documents successful therapeutic removal using office-based needle probing after visualization.

Limitations:

Single Case Report: Limits generalizability and statistical power.
Retrospective Nature: Potential for recall bias.
Lack of Initial Imaging: Absence of ultrasound or radiography prior to FB removal limits objective documentation of its location (Note: Clarify X-ray timing in Case Presentation).
Needle Probing Caveat: Use of needle probing as a diagnostic tool without prior suspicion/imaging is not standard of care and carries risks. Its applicability is context-specific.
Scope: Limited discussion of potential complications (e.g., infection, granuloma) or broader differential diagnoses due to case specificity.
Patient Perspective: The patient was a physician who self-diagnosed and treated, which may not reflect typical patient behavior. If you have any foot-related diseases, it's best to consult a specialist as soon as possible.

## Patient consent

Written informed consent was obtained from the patient for publication of this case report and accompanying images. A copy of the written consent is available for review by the Editor-in-Chief of this journal on request.

## Prior presentation

This case has not been previously presented in any form.Preprint.

## Preprint

A previous version of this manuscript was posted on the Qeios preprint platform prior to submission and is available under the DOI: https://doi.org/10.32388/26P5CL.

## Journal consideration

This manuscript is not under consideration by any other journal.

## Sources of funding

This research did not receive any specific grant from funding agencies in the public, commercial, or not-for-profit sectors.

## Ethics approval

This study was approved by the Ethics Committee of Taizhou Hospital, Zhejiang Province, China. All procedures followed institutional ethical guidelines and adhered to the principles of the Declaration of Helsinki.

## Author contribution

1. Huabin Chen

study concept or design, data collection, data analysis or interpretation, writing the paper, others, all

2. Feng Zhang: Writing – review & editing.

## Guarantor

Huabin Chen, Authors himself,who accept full responsibility for the work and/or the conduct of the study, had access to the data, and controlled the decision to publish.

## Research registration number

Research registry 11336

## Conflict of interest statement

The authors declare no competing interests.

## Data Availability

All data supporting this study are included within the manuscript.
